# Whole-Genome Sequencing of Micrococcus luteus MT1691313, Isolated from the Mariana Trench

**DOI:** 10.1128/MRA.00369-21

**Published:** 2021-06-10

**Authors:** Zhen Wang, Ji-xing Feng, Xue-peng Li, Jian Zhang

**Affiliations:** a School of Ocean, Yantai University, Yantai, China; University of Southern California

## Abstract

Micrococcus luteus MT1691313 is a Gram-positive bacterium isolated from the deep-sea sediment located at a 4,448-m depth in the Mariana Trench. Here, we report the complete genome sequence of this strain, which has a genome size of 2.32 Mb with a GC content of 72.04%.

## ANNOUNCEMENT

Deep-sea environments are some of the most extensive extreme environments on Earth and are characterized by high hydrostatic pressure, low temperature, low oxygen, and absence of light (1). Micrococcus luteus is a high-GC-content, Gram-positive, strictly aerobic coccus and has been isolated from a wide range of environments, such as air, soil, water, and skin (2–5). In this study, a bacterium named M. luteus MT1691313 was isolated from the surface sediment sample (Mariana Trench, 4,448-m depth, 11.327°N, 142.188°E) obtained by a gravity sampler on the research vessel *Xiangyanghong 09* during the cruise of September 2016. Here, we report the complete genome sequence of M. luteus strain MT1691313 to better understand the potential for its industrial application.

Strain MT1691313 was isolated using marine 2216E agar medium (Haibo, China) as described previously (6). A single colony was picked and cultured for 16 h at 28°C in marine 2216E medium; genomic DNA extraction, 16S rRNA gene amplification, sequencing, and sequence analysis were performed as described previously (6), revealing that strain MT1691313 shared the highest 16S rRNA gene sequence identity (99.35%) with M. luteus strain 1872. For genomic sequencing, a single-molecule real-time (SMRT) sequencing library was constructed with an insert size of 10 kb using the SMRTbell template kit v1.0, and an Illumina sequencing library was generated using the NEBNext Ultra DNA library prep kit for Illumina (New England BioLabs [NEB], USA). The whole genome of MT1691313 was sequenced using the PacBio Sequel and Illumina NovaSeq PE150 platforms, and totals of 49,618 (*N*_50_, 13,332 bp; ∼214× genome coverage) and 14,400,443 (∼989× genome coverage) reads were obtained, respectively. The low-quality PacBio reads were filtered (<500 bp), the long reads were selected (>6,000 bp) as the seed sequence, and the other shorter reads were aligned to generate one contig without gaps using SMRT Portal assembly software v5.0.1 (7). The preliminary assembly result was corrected by the variant Caller module of the SMRT Link software (v5.0.1) (7) using Illumina reads aligned by Burrows-Wheeler Aligner (BWA) v0.712 (8). Based on the overlap between the head and the tail, the chromosomal sequence was confirmed to form a circle, and then the initial site was corrected using BLAST with the DNAa database (https://www.ncbi.nlm.nih.gov/genome/?term=DNAa) (9). The genome component and gene functions were further predicted using the NCBI Prokaryotic Genome Annotation Pipeline (PGAP) (10). Secretory proteins were predicted with SignalP v4.1 (11). Default parameters were used for all software unless otherwise specified.

The complete genome of M. luteus MT1691313 consists of a circular chromosome of 2,437,056 bp with a G+C content of 73.04%, and no plasmids were identified. A total of 2,130 protein-coding genes, 48 tRNAs, 6 rRNAs, 3 noncoding RNAs (ncRNAs), and 53 pseudogenes were identified. Genes encoding extracellular lipase and amylase, i.e., phospholipase, lipase, and alpha-amylase, were also detected. The complete genome sequence reported here may facilitate the development and application of deep-sea bacteria in industry.

### Data availability.

The 16S rRNA gene and complete genome sequences have been deposited in GenBank under the accession numbers MZ156964.1 and CP072656.1, respectively. The raw reads were deposited in the Sequence Read Archive (SRA) under the accession numbers SRR14532941 (PacBio) and SRR14125858 (Illumina).
